# Attention to News Media, Emotional Responses, and Policy Preferences about Public Health Crisis: The Case of Fine Dust Pollution in South Korea

**DOI:** 10.3390/ijerph182413325

**Published:** 2021-12-17

**Authors:** Soohee Kim, Yong-Chan Kim

**Affiliations:** Department of Communication, Yonsei University, Seoul 03722, Korea; soohkim@yonsei.ac.kr

**Keywords:** news attention, emotion, policy preferences, public health crisis

## Abstract

This study examines how attention to science and political news may influence the way people feel about an environmental risk, and how this in turn impacts policy preferences. Using an online survey conducted on the issue of fine dust pollution in South Korea, this study found that science news attention was associated with greater anxiety and anger about the issue, whereas political news attention was associated with fear and sadness/depression (as well as anxiety and anger). Furthermore, mediation analysis showed that science news attention indirectly influenced support for preventive policy through anxiety, whereas political news attention indirectly influenced punitive policy support through anger and fear. Theoretical and practical implications of this study are discussed.

## 1. Introduction

Environmental risks such as air pollution have been increasingly recognized as one of the major public health concerns [1,2]. Accordingly, news media have addressed these issues not only in the domain of science or the environment but also in the domain of politics because the government is obligated to hold air polluters accountable [3,4]. The political coverage of environmental risks, however, has often raised concerns among experts since it appears to trigger negative responses among the public [5,6]. Researchers have suggested that delivering stories about environmental problems as political news (not as scientific news) increases incorrect perceptions about environmental issues [7], generates negative feelings among the audience such as helplessness, anger, and fear [8,9], and discourages public engagement with policymaking processes that deal with the issues [10]. That is, telling stories about science or environmental crises as political news may not only distort scientific discussions about the issues [11] but also lead to affective responses that can negatively affect public judgment and behavior. 

Although this line of research indicates that specific use of news media genres (e.g., political news) may be distinctively linked to certain negative affective responses, potentially discouraging people from forming accurate perceptions about the risk or engaging in actions to mitigate the risks, few empirical studies have investigated how specific patterns of media use may be associated with certain judgments about the risks, considering the role of affective mechanism in the processes. Instead, previous work has primarily focused on the consequences of specific coverage of an environmental crisis such as increased skepticism and decreased motivation to engage with the crisis [10]. However, given the diverse media coverage these environmental or health risks receive and its potential influence on public responses, it is important to examine whether specific media use is linked to distinct affective responses to certain health/environmental risk issues and judgement about the risks.

Therefore, the goal of this study is twofold. First, we examine how specific use of news media (science and environment news vs. political news) is associated with discrete emotions felt toward an environmental issue. Second, we assess the role of discrete emotions in mediating the influence of media use on policy preferences regarding the issue. Grounded in the literature on news media effects and theories of discrete emotion, we investigate how discrete emotions, associated with specific use of media, may influence people’s judgment about important environmental/health risks. To the best of our knowledge, this study is the first to examine the genre-specific effects of news media use on public judgment about an environmental risk while simultaneously testing the affective mechanism underlying the process. We address this question in the context of the fine dust problem in South Korea where the issue of fine dust pollution has been a critical social problem and has received extensive media attention. By delineating the association of specific patterns of media use, discrete emotional responses to a risk issue, and preference for measures related to how to address the issue, this study sought to provide insights into how media coverage on environmental or health risk issue can shape public judgment or behavior in times of crises.

## 2. Literature Review

### 2.1. The Context of Fine Dust Pollution Problem in South Korea

The issue of fine dust pollution has been a critical social problem in South Korea. The air quality in South Korea ranked 173rd out of 180 countries [12]. People think fine dust pollution is one of the most important issues facing the country and that 97% of Koreans believe fine dust is a serious threat that causes them physical or psychological pain [13]. Indeed, the issue of fine dust is now considered a social disaster in South Korea [14].

Importantly, the issue of fine dust pollution has been considered a complicated political issue with implications for both international (e.g., relationships with China) and domestic politics (e.g., conservative parties attacking a president from the liberal party for cozying up with China). Accordingly, the fine dust problem has received extensive media attention in South Korea [15]. However, the news media of fine dust have been consistently criticized for their emotional, fear-mongering coverage of the issue. It has been noted that news reports focus on who is responsible for the problem or how devastating the results would be—instead of providing systematic analysis of its causes and possible solutions [16,17]. Scholars and experts pointed out that news coverage features a sensational, emotion-triggering news style to harness public attention [18]. 

Existing empirical evidence suggests that news coverage of fine dust increases negative affective responses. For example, a study based on content analysis of South Korean news media found that news media tends to attribute the primary cause of fine dust pollution only to external factors (e.g., air pollutants generated in China) [15], not internal factors (e.g., domestic source of pollution). Because inferences of responsibility in others for a negative outcome can trigger anger [19,20], news coverage of fine dust that emphasizes the responsibility of external factors may evoke anger about the situation. Other reports also indicate that political news depicts the fine dust problem as a life-threatening one that leads to irrevocable health consequences, but present the public with temporary and individual-based measures that are seemingly ineffective to reduce the hazardous health consequences of fine dust exposure, which may result in fear and depression [17]. Since political news tends to focus on political implications of social issues, it may highlight stories such as who is accountable for the problem (e.g., government, certain political parties) while featuring political actors as official sources for the issue [21]. This tendency, as a result, may lead to lower accuracy and less systematic coverage of the issue. Meanwhile, it has been shown that science and environmental news provides a more balanced and systematic coverage of the problem. For example, regarding the causes of fine dust pollution, these reports provide the public with information about the various possible causes showing that domestic factors (e.g., car exhausts)—not just external factors (e.g., China)—can contribute to the fine dust problem [22,23]. 

Taken together, news coverage on fine dust pollution in South Korea points toward the possibility that attending to political or science news is distinctively associated with emotional reactions to the issue of fine dust, thus affecting subsequent judgment of how to address the risk. This study explores this possibility by examining the relationships between attention to science/environment and political news, discrete emotions about the fine dust problem, and preferences for preventive or punitive policies. 

### 2.2. News Media Attention and Public Responses to Risks

Scholars have consistently examined questions regarding how news media effects should be measured. While many studies have focused on measures based on time such as time spent on the media or frequency of media use [24,25], research has increasingly used measures that can embrace individuals’ activity in media use. For example, the literature on the audience activity model of media effects [26] proposes that media use is not passive as it is essentially driven by individuals’ prior interests and motivation [27]. This research suggests that the extent to which individuals attend to or elaborate on given information is critical in predicting the impact of media [28]. That is, whether and how individuals attend to specific types or contents of media can exert a powerful influence on how they think about or make judgments about important social issues. Even though there have been some debates regarding the conditions or contexts in which media attention can change those perceptions and judgments [29], it is now generally believed that the construct of media attention provides a better explanation of how media influence individuals’ perceptions, judgments, and behaviors [30,31,32].

Extant research examining the relationships between media use and public responses to risk and social issues has shown that attending to distinct genres of news media changes the way individuals react to those issues. For example, Zhao et al. [33] examined how attention to science or political news is related to perceptions of risk and policy support in the context of global warming. They found that attending to science news promotes beliefs consistent with scientific views while attending to political news increases incorrect perceptions and beliefs. Similarly, Namkoong et al. [34], noting that attention is more closely associated with individuals’ cognitive and emotional responses than other variables such as exposure, examined how attention to different genres of news (e.g., TV and newspaper) is linked to political campaign participation. Their findings showed that the levels of news media attention are distinctively related to discrete emotions, resulting in different levels of participation. Taken together, these studies indicate that attending to specific genres of news, by leading individuals to learn certain issues through the lens of distinct perspectives or frameworks, can lead to different judgments and behaviors regarding the issues.

In line with this research, the current study also examines the relationships among media use, emotional responses, and policy preferences focusing on the extent to which individuals attend to specific genres of media—that is, attention to political or science/environment news. As discussed earlier, as a complicated form of political and environmental issue that has been highly politicized and differentially covered in political and science/environment news, the issue of fine dust pollution provides an ideal context in which to examine the relationships between news attention, emotions, and judgments. Whether individuals attend to science or environment-related news or political news in general is likely to be distinctively linked to specific emotional responses to the issue, thus shaping subsequent judgment or behavior in the context of risks. To do so, we focus on specific emotions that are likely to be relevant to the risk issue, namely anger, fear, anxiety, and sadness. We chose these intense emotions not only because they are associated with clear action tendencies and goals [20,35] but also because they are most frequently triggered when individuals encounter certain threats, including public environmental or health crisis [36].

### 2.3. News Media Attention and Emotional Responses

While media and communication literature have long focused on cognition as a mediator of media effects [37], scholars have increasingly examined emotion to better understand the effects of media on individuals [38]. Considering that emotions exert powerful influence on subsequent judgments and behaviors, studies examining the mechanism underlying the impact of media have focused on the role of emotion in the process [39,40]. Various theories of emotion suggest that different types of information or news can trigger distinct emotional responses by affecting their cognitive appraisal of the situation. For example, according to cognitive appraisal theories of emotion, each emotion can be defined by central dimensions (e.g., responsibility, certainty, control) and that these cognitive dimensions differentiate one’s emotional experience and its action tendencies [20]. The theory suggests that anger is associated with the belief that another person is responsible for a negative consequence. Thus, anger tends to elicit behavior aimed at punishing those who are responsible for a problem [19]. On the other hand, anxiety is related to the state of uncertainty and tends to elicit behavior that can resolve the uncertain negative outcome (e.g., seeking information about how to deal with the negative consequences) [19,20]. Fear, while closely related to the feeling of anxiety (e.g., appraisal of uncertainty), has been distinguished from anxiety in the sense that it is evoked when individuals believe they have little control over the situation [19]. Thus, fear is more associated with the desire to avoid future harm and escape the situation immediately than anxiety [19,20,41]. 

Nabi’s cognitive-functional model also explains how discrete emotions may affect subsequent information processing. According to Nabi [39,42], discrete emotions differentially influence the manner of information processing depending on the extent to which individuals experience motivated attention (i.e., motivated to engage with message) and motivated processing (i.e., motivated to achieve goal associated with emotions). For example, fear discourages individuals from engaging with the affect-inducing situation and subsequently reduces the motivation to carefully process the information. Anger, on the other hand, encourages engaging with the situation and increases the motivation to carefully process the information. While Nabi’s work [39,42] mostly focused on the context of persuasion and the effects of specific message-induced emotion, it suggests that discrete emotions can change how individuals understand the given situation, thus shaping subsequent judgment and behavior.

Based on these theories and research, we predict that attention to different news media may cultivate distinct emotions about important social issues among their audiences [5,43]. First, we expect that individuals who attend to news media—regardless of whether it is science/environmental or politics—are likely to feel anger and anxiety about the fine dust problem. As discussed earlier, news reports on fine dust pollution in Korea in general emphasize the role of China as the source of Korea’s pollution [15]. Therefore, the perception of responsibility attributed to China, with the perception that it unfairly causes health problems among people in Korea (the core relational theme of anger), will lead people to feel angry. In addition, these individuals may feel anxiety about the fine dust problem. Studies indicate that news media use in general is positively related to understanding of science or health issues [44]. Thus, the heightened understanding of the fine dust problem, by increasing awareness of related threats and their susceptibility to those threats (the core relational theme of anxiety) [45], might lead people to feel anxiety about problematic states [46]. 

We expect that political news use may trigger additional negative emotions such as fear and sadness. As discussed earlier, fear arises when a goal has been threatened and the individual believes he or she has little power over the situation [19]. Since one of the prominent features of political coverage of fine dust in Korea is to focus on depicting critical negative health consequences without providing information about how to mitigate the problem, attending to political news is likely to lead to fear about the fine dust problem. In addition, political news use is likely to evoke sadness/depression. Since fine dust pollution is often described in political news as a problem that is caused by contextual/external factors (e.g., China) and causes serious and irrevocable health consequences (the core relational theme of sadness), individuals who attend to political news are more likely to feel sadness/depression [19]. On the other hand, science news attention may or may not be associated with fear and sadness. Considering that science news leads people to understand the threats associated with the risks and their susceptibility to the threats [47], science new use may evoke fear and sadness. However, as science news tends to provide a more systematic coverage of risk issues, for example offering people information about how to prevent or address the risks, science news use may not be necessarily linked to fear or sadness. Therefore, we examine this as a research question. 

### 2.4. News Media Attention and Policy Preferences

Beyond the influence of news media on emotional responses, news media use may directly impact individuals’ decisions to support specific types of policy. Studies have found that news attention has a powerful influence on individuals’ beliefs and judgments about social issues [33,48]. In particular, this study examines whether specific news media attention is related to differential policy preferences—preventive versus punitive policies. These policy preferences represent substantially different judgment of how to address a given problem [49,50]. For example, preventive policies include measures that can be taken to prevent the problems, while punitive policies include measures that focus on punishing the individuals that are seen to be responsible for the problem. Specifically, we make two predictions about the relations between genre-specific news attention and policy preference. First, we predict that attending to science news increases support for both preventive and punitive policies to address the risk. As scientific news use increases the scientific knowledge and understanding of its viewers [47,51], individuals who are aware of the threats associated with the risk may support various policies that can help reduce the problem [52]. Increased understanding about scientific issues may lead people to choose policies that are more fundamental and have long-term effects such as preventive policy as well as punitive policy [53,54].

On the other hand, attending to political news is likely to increase support for punitive policy, but not preventive policy. As political news focuses on depicting destructive consequences of the fine dust problem without providing information about what individuals can do to solve the problem, people will remain uncertain about how to deal with the problem. This confusion and heightened uncertainty constructed by political news is likely to motivate people to reduce their uncertainty and perceptions of threat, which may lead them to support measures that can show more direct, visible effects, such as punishing those who seem to be at fault (as opposed to preventive measures that takes longer time to see the effects). While not in the context of environmental risks, Gardarian [8] also found that the media’s emphasis on threatening information and evocative imagery boosted support for punitive policy, as threatening news stories lead people to prefer policy that is matched with fear-inducing cues. 

### 2.5. Emotion and Policy Preferences

Empirical evidence suggests that discrete emotions play a crucial part in the formation of judgments and preferences [34,38]. As people use their emotional reactions to a stimulus as a source of information in making judgments about that stimulus, different emotional reactions to risk issues give rise to different judgment and behavior about the risk. In particular, the literature on risk communication has shown that discrete emotions exert unique influence on perception and judgment in the context of risks. For example, Smith and Leiserowitz [36] examined the impact of discrete emotion on policy support in the context of global warming and found that discrete emotions such as worry (anxiety) and hope were more strongly related to increased policy support than other variables such as cultural worldviews, general negative affect, or sociodemographic variables. 

More relevant to the present study, Bohm and Pfister [55] examined how the perception of a risk is associated with specific types of action tendencies, mediated by emotions. Specifically, they proposed to classify emotions into ethical (e.g., anger, outrage, or guilt directed to particular agents) and loss-based emotions (e.g., fear, worry, or sadness based on the anticipated negative consequences), arguing that environmental risks are evaluated substantially differently depending on these emotions. Their findings showed that ethical emotions were associated with a tendency to aggression/retaliation whereas loss-based emotions were associated with a tendency to help/prevention. Xie et al. [56] also examined the role of discrete emotions in public reactions to different types of risk (i.e., technological and natural hazards). Based on the cognitive appraisal perspectives of emotion, they suggested natural hazards such as earthquakes could be considered as inevitable, whereas technological hazards could be thought to be more controllable. They argued that, because technical hazards and natural hazards are critically different in “the perception of controllability and blameworthiness” (p. 451), these appraisals could induce distinct emotional reactions, which then lead to different public reactions. Indeed, their findings showed that discrete emotions (e.g., anger, fear, regret) differentially mediated the influence of TV use on support for preventive and punitive strategies. Even though their focus was on the influence of the different types of risk, the findings demonstrate that discrete emotions, induced from distinct appraisals of the situation, lead to differential judgments and policy preferences regarding the risk.

Based on these findings from previous studies, the present study predicts that anxiety, which arises from the appraisal that the situation is threatening and uncertain [19], may increase preference for preventive policies—the policies that can provide protection from the threat. Previous research shows that anxiety encourages people to consider measures that have preventive effects [40]. On the other hand, anger, stemming from the perception that others have caused the problem and that it unfairly causes problems to oneself [19], might evoke the desire to retaliate against those responsible for the given problem, thus increasing support for punitive policies [19]. Consistent with this prediction, research found that anger was strongly related to support for retribution-focused policy [40]. Likewise, fear, stemming from the perception that the threat is highly relevant and significant, might trigger the desire to immediately address the problem. As fear leads individuals to be less optimistic about the future [57], fearful individuals may support for measures that can have more immediate, visible effects such as punishment. 

It is more complicated to predict the influence of sadness on policy support. On the one hand, it is possible that sadness leads to support for protection-focused policy since it alerts individuals to seek comfort [58], leading to a preference for supportive measures [59]. Yet, it is also possible that sadness increases preference for punitive policies insofar as it increases feelings of sympathy for potential victims (e.g., individuals) while simultaneously increasing feelings of condemnation for those who might have caused the problem (e.g., government, industry) [50]. Therefore, we investigate the relationship between sadness and policy preferences as a research question. 

We expect that these emotions mediate the influence of genre-specific news attention on policy preferences. To the extent the preventive policies are considered to be policies that provide protection from the threat, science news that describes how the problem poses potential threats to oneself—the core relational theme of anxiety—is likely to increase preference for preventive policies through the feeling of anxiety. On the other hand, political news that emphasizes who is more or less responsible for the problem may elicit anger, which then may increase the support for policies that focus on punishment [59,60]. Likewise, political news that emphasizes the severity of a potential threat of a given risk (without providing information about how to solve or mitigate the problem) is likely to lead to fear, thus increasing the preference for measures that can produce more immediate effects, such as punitive policies. Thus, in line with previous work suggesting that emotion mediates the effects of media use on individuals’ attitudes and behavior [61], we expect that discrete emotions may mediate the impact of news attention on policy preferences. 

Below is the summary of hypotheses and research questions.

**Hypothesis** **1** **(H1).** *Science news attention will be positively associated with (a) anxiety and (b) anger about the fine dust problem.*

**Hypothesis** **2** **(H2).** *Political news attention will be positively associated (a) anxiety, (b) anger, (c) fear, and (d) sadness about the fine dust problem.*

**Hypothesis** **3** **(H3).** *Science news attention will be positively associated with support for (a) preventive policies and (b) punitive policies.*

**Hypothesis** **4** **(H4).** *Political news attention will be positively associated with support for punitive policies.*

**Hypothesis** **5** **(H5).** *Anxiety will be positively associated with support for preventive policy.*

**Hypothesis** **6** **(H6).** *(a) Anger and (b) fear will be positively associated with support for punitive policy.*

**Hypothesis** **7** **(H7).** *The influence of science news attention on support for preventive policy is mediated by anxiety.*

**Hypothesis** **8** **(H8).** *The influence of political news attention on support for punitive policy is mediated by (a) anger and (b) fear.*

**RQ1:**  *Will science news attention be positively associated with fear or sadness about the fine dust problem?*

**RQ2:**  *Will sadness be positively associated with support for preventive or punitive policies?*

**Figure 1:**
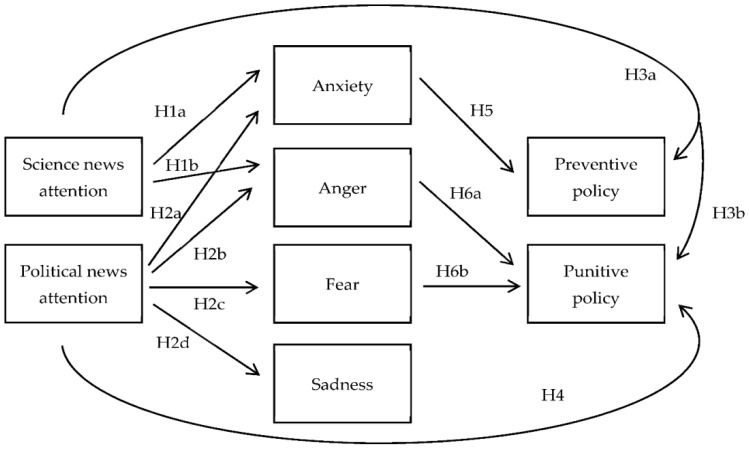
Conceptual model of the study.

**Figure 2:**
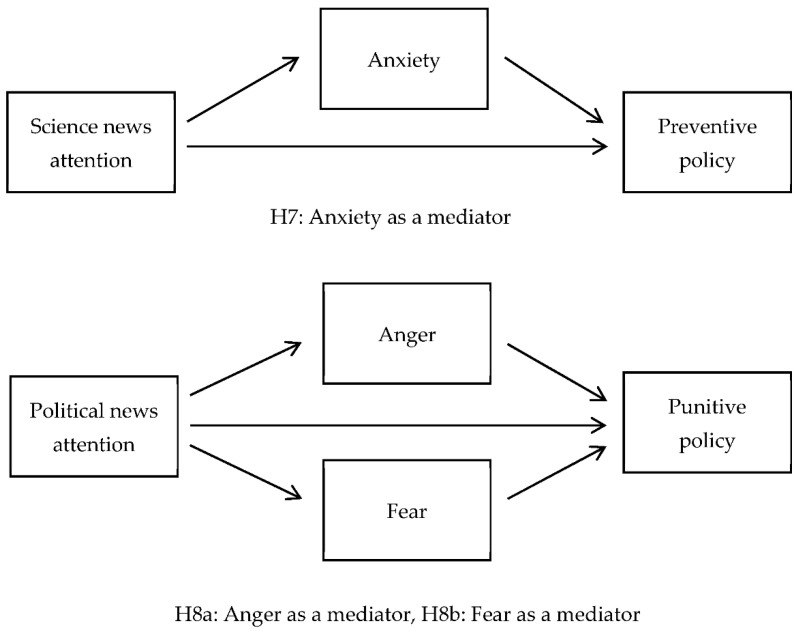
Hypothesized mediation model.

We propose a conceptual model to describe the potential relationships between news attention, emotional responses, and policy preferences (Figure 1). In addition, we present a mediation model of this study (Figure 2).

## 3. Method

### 3.1. Sample

To test our hypotheses and research questions, an online survey was conducted between 20 February and 26 February 2019. Survey respondents were recruited from the online panel directory of a research firm in Korea that was highly regarded for its systematic survey execution and high-quality outcomes. The survey firm provided a panel of nationally representative respondents. The panel consisted of individuals who were willing to participate in the survey. The research firm sent emails that included the link to the survey to the panel members. Panel members received monetary incentives if they participated and completed the survey. We used a cluster sampling procedure with three criteria: (1) gender, (2) age (20s, 30s, 40s, 50s and older), and (3) the main regions of South Korea (Seoul/Incheon/Gyeonggido, Gangwondo, Choongcheongdo, Gyeonngsangdo, and Jeonrado/Jejudo). An email invitation was sent to 6353 potential respondents and 1560 respondents opened the email. Out of 958 who actually participated in the survey, 510 respondents completed the survey (within-panel completion rate: 33%). About 49% of the respondents were female, and the mean age of respondents was 41.1 (SD = 12.6 years). 

### 3.2. Measures

To measure attention to science/environment and political news, respondents were asked the degree to which they attend to each news media. Based on the measures employed in previous studies [26,37,51], respondents were asked, “How closely do you follow news about each of the following?” The indicators for science and environmental news attention included two items: (a) science and technology and (b) the environment. Responses were measured on a 5-point scale ranging from 1 (not at all) to 5 (very closely) (α = 0.73, *M* = 3.63, *SD* = 0.85). The indicators for political news attention included four items: (a) national politics, (b) local politics, (c) world politics, as well as (d) government, political parties, and politicians. Responses to these four items were measured on the same 5-point scale (α = 0.86, *M* = 3.46, *SD* = 0.83). 

Emotional responses were measured by asking respondents “How strongly do you feel each of the following emotion, when thinking about the issue of fine dust?” Their responses were measured on a 5-point scale ranging from 1 (not at all) to 5 (strongly): anxiety (*M* = 4.21, *SD* = 0.90), anger (*M* = 3.78, *SD* = 1.01), sadness (*M* = 3.41, *SD* = 1.13), and fear (*M* = 3.70, *SD* = 1.10).

To measure policy preferences, respondents were asked about their support for prevention-focused policies and retribution-focused policies. Indicators for prevention-focused policies included items such as establishing a research fund for mitigating fine dust problem, giving incentives to companies that install facilities designed to reduce fine dust, collaborating with foreign countries to address the fine dust pollution, and making provisions to reduce fine dust problem. Indicators for retribution-focused policies included items such as penalizing companies and transportation systems that emit pollutants that cause the fine dust pollution and prosecuting business shutdown on companies that do not follow guidelines. Respondents were asked, “How much would you support each of the following policy?” Responses were measured on a 5-point scale ranging from 1 (not at all) to 5 (strongly support). Thus, two policy preference indices—support for prevention-focused policy (α = 0.81, *M* = 4.13, *SD* = 0.62) and support for retribution-focused policy (α = 0.79, *M* = 3.93, *SD* = 0.69)—were created.

Control variables included prior beliefs and interest in the fine dust issue as well as socio-demographic variables. Specifically, perceived risk (*M* = 5.89, *SD* = 1.12) and interest in the fine dust problem (*M* = 4.23, *SD* = 0.75) as well as age (*M* = 41.1, *SD* = 12.6), gender (male = 51.2%), education (high school or less: 24.9%; college or bachelor’s degree: 64.5%; more than bachelor’s degree: 10.6%), and political orientation (ranged from 1–5, a higher score indicating more conservative attitudes; *M* = 3.65, *SD* = 1.12) were measured and controlled in the analyses.

### 3.3. Analysis Strategy

Prior to testing the proposed model, a correlations analysis was conducted to examine the relationships between the variables of interest (Table 1). To investigate the relationships between news media attention, emotional reactions, and policy choice, we ran a path analysis using a structural equation model, using STATA 14.0. In addition, to confirm the mediation relationships suggested in the path analysis, independent tests of significance of indirect effects were also conducted, employing a series of bootstrapping and bias-corrected confidence intervals analyses [62].

## 4. Results

**Figure 3:**
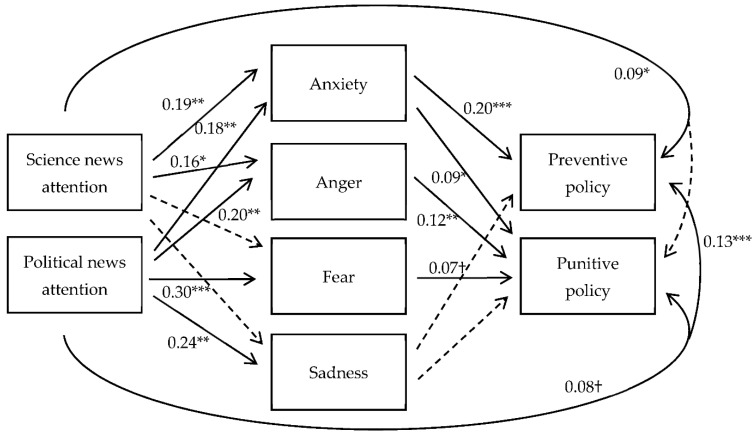
Results of hypothesized model. Note. Standardized coefficients are shown. The effects of control variables on emotions and policy support were estimated but not depicted here. † *p* < 0.10, * *p* < 0.05, ** *p* < 0.01, *** *p* < 0.001.

The results of the path analysis are summarized in Figure 3. Upon conducting the preliminary analysis, the insignificant paths from the sociodemographic variables were excised from the analyses. The model produced the following indices, suggesting a good fit with the data: χ^2^(2) = 2.24; *p* = 0.33; CFI = 1.00, TLI = 1.00, RMSEA = 0.02. Of note, political conservatism was negatively related to political news attention (*b* = −0.09, *SE* = 0.03, *p* < 0.01) and science news attention (*b* = −0.10, *SE* = 0.03, *p* < 0.01). Educational attainment was positively related to both political news attention (*b* = 0.12, *SE* = 0.04, *p* < 0.01) and science news attention (*b* = 0.08, *SE* = 0.04, *p* < 0.05). Being female was negatively related to political news attention (*b* = −0.22, *SE* = 0.07, *p* < 0.01) but not associated with science news attention (*b* = −0.10, *SE* = 0.07, *p* = 0.18). Women were more likely to feel all of the emotions (anxiety: *b* = 0.35, *SE* = 0.08, *p* < 0.001; anger: *b* = 0.22, *SE* = 0.09, *p* < 0.05; sadness: *b* = 0.41, *SE* = 0.09, *p* < 0.001; fear: *b* = 0.54, *SE* = 0.09, *p* < 0.001). Political conservatism was negatively related to support for both preventive policies (*b* = -.05, *SE* = 0.02, *p* < 0.05) and punitive policies (*b* = -.07, *SE* = 0.02, *p* < 0.01), which made sense when considering the fact that the liberal party was the ruling party in South Korea. Perceived threat was positively related to political news attention (*b* = 0.30, *SE* = 0.04, *p* < 0.001) as well as science news attention (*b* = 0.32, *SE* = 0.04, *p* < 0.001). Interest in the fine dust issue was also positively related to both political news attention (*b* = 0.34, *SE* = 0.05, *p* < 0.001) and science news attention (*b* = 0.35, *SE* = 0.05, *p* < 0.001).

First, we examined how people felt different emotions toward the fine dust problem depending on their use of news media. Consistent with our expectation, the results showed that science news attention was positively and significantly associated with feeling anxiety (*b* = 0.19, *SE* = 0.06, *p* < 0.01) and anger (*b* = 0.16, *SE* = 0.06, *p* < 0.05), thus providing support for H1a and H1b. Regarding RQ1, attention to science news was associated with neither fear (*b* = 0.10, *SE* = 0.07, *p* = n.s.) nor sadness (*b* = 0.11, *SE* = 0.07, *p* = n.s.). On the other hand, attention to political news was related to increased feelings of anxiety (*b* = 0.18, *SE* = 0.06, *p* < 0.01), anger (*b* = 0.20, *SE* = 0.07, *p* < 0.01), fear (*b* = 0.30, *SE* = 0.07, *p* < 0.001), and sadness (*b* = 0.24, *SE* = 0.07, *p* < 0.01) (H2d), confirming H2a–d. 

Regarding the association of news attention and support for different policy initiatives, the results show that science news attention is positively related to support for preventive policy (*b* = 0.09, *SE* = 0.04, *p* < 0.05) but not for punitive policy (*b* = 0.05, *SE* = 0.04, *p* = 0.20), providing support only for H3a. In addition, to a marginal extent, political news attention was positively related to support for punitive policy (*b* = 0.08, *SE* = 0.04, *p* = 0.07), supporting H4. However, differing from our expectation, political news attention was also associated with preventive policy support (*b* = 0.13, *SE* = 0.04, *p* < 0.001).

As hypothesized, anxiety was positively and significantly associated with support for preventive policy (*b* = 0.20, *SE* = 0.04, *p* < 0.001). Thus, H5 was supported. However, the results showed that anxiety was also linked to support for punitive policy (*b* = 0.09, *SE* = 0.04, *p* < 0.05). Anger was positively related to support for punitive policy (*b* = 0.12, *SE* = 0.04, *p* < 0.01) but not for preventive policy (*b* = 0.02, *SE* = 0.04, *p* = *n.s.*), confirming H6a. In support of H6b, fear was positively associated with support for punitive policy, even though it did not reach statistical significance (*b* = 0.07, *SE* = 0.04, *p* = 0.09). Furthermore, RQ2 was to examine the relationship between sadness and policy preferences. The results indicated that sadness was associated with support for neither preventive policy (*b* = 0.06, *SE* = 0.04, *p* = n.s.) nor punitive policy (*b* = 0.06, *SE* = 0.04, *p* = n.s.), suggesting that the feeling of sadness is not linked to these policy preferences.

Next, based on the results of path analyses, we investigated how discrete emotions would mediate the influence of news attention on policy preferences. To formally test the mediating role of emotional responses in the impact of news use, independent tests of significance of indirect effects were conducted using a series of bootstrap tests, with 5000 trials and bias-corrected 95% confidence intervals analyses [62]. As shown in Table 2, the analyses revealed the indirect effects of science news attention on preference for preventive policy through the feeling of anxiety (*b* = 0.08, *SE* = 0.02), supporting H7. In addition, the results showed that both anger (*b* = 0.08, *SE* = 0.02) and fear (*b* = 0.08, *SE* = 0.02) mediated the influence of political news attention on support for punitive policy, confirming H8a,b. Given the results of previous analyses that indicated that political news attention was positively related to support for preventive policy, we conducted additional mediation tests in which anxiety mediated the influence of political news use on support for preventive policy. The results confirmed the role of anxiety in mediating the relationship between political news attention and preference for preventive policy (*b* = 0.07, *SE* = 0.02). 

## 5. Discussion

The primary goal of this study was to investigate the relationship between news media use and discrete emotions felt toward a health risk issue, and the role of these emotions in mediating the influence of media on policy preferences regarding the issue. Drawing on media effects research and theories of discrete emotion, we hypothesized that the extent to which people attend to specific genres of news media will exert an influence on public judgment about health risk, mediated by emotional responses. 

Our findings indicate that attention to different genres of news media—political vs. science news—is indeed associated with distinct emotional responses about the environmental risk; whereas science and environmental news attention was associated with greater anger and anxiety about the fine dust problem, political news attention was associated with feelings of fear and sadness as well as anger and anxiety. First, the finding that both political news and science news attention elicit greater feelings of anxiety and anger indicates that news media attention—regardless of the specific genre—has the potential to lead to stronger affective responses about the risk. As previous studies show that increased involvement with an issue leads individuals to perceive greater threats associated with the issue [51], it is possible that by increasing awareness and involvement with important social risks, news media attention triggers greater affective responses such as anger [63] and anxiety [64] about the risk issue.

Perhaps more importantly, the findings reveal that only political news media attention is associated with greater feelings of fear and sadness. Considering that perceptions of uncontrollability and seriousness of the risks lead people to feel extreme fear about the issue [19], it is possible that political news media have emphasized the uncontrollable aspects of the threat associated with the environmental risk, thus eliciting fear among the public. Furthermore, political news might have induced a feeling of sadness/depression, by implying that individuals cannot cope with the problem (as situational forces are responsible for the problem). Taken together, these findings seem to be in line with previous work that cautions against political coverage of science [7]; political news use may cultivate greater negative affective responses, which might be unhelpful and disruptive in crisis situations [5].

The findings also show that distinct emotions evoked by attending to specific news genres are indeed differentially related to policy preferences. First, anger and fear were positively associated with preference for punitive policy, consistent with previous work that showed anger to predict retaliation-related behavior [65] and fear to increase support for punitive measures [66]. These results suggest that feeling anger and fear about the risk are likely to lead people to prefer retaliation-focused policies to prevention-focused policies—which eventually becomes a critical barrier for implementing long-term policy [50]. 

Anxiety, on the other hand, promoted support for preventive as well as punitive policies. While this finding contradicts our initial prediction that anxiety will increase support for preventive measures and not punitive measures, alternative explanations seem possible. For example, it is possible that the level of perceived threat might have played a role in the process. When individuals perceive an extreme threat, anxiety may lead people to consider various potential measures (regardless of the preventive or punitive nature of measures), while it may lead them to choose preventive measures when they perceive a minimal to moderate threat. Likewise, the domain of issues might have also influenced the findings. Some prior work found that anxiety increased support for aggressive military action against terrorists [46] whereas other research showed that anxiety increased the support for punishment of criminals [67]. Therefore, more research is needed to identify various potential factors that influence the behavioral effects of anxiety in different contexts. The findings also indicated that sadness was not associated with any policy support. Although this is consistent with prior work that has characterized sadness as an inaction-oriented emotion [18], more research is warranted considering studies that suggest the behavioral impact of sadness, such as promoting the desire to seek out protection-focused measures [68].

Beyond the mediating role of discrete emotions on policy support, news media attention was directly associated with greater support for policies aimed to reduce the threat of health risks. First, the finding shows that attending to science and the environment news increases support for preventive, but not punitive policy. People might have developed better understandings about science issues while attending to science and environmental news, thus becoming more supportive of policies that can have more fundamental effects, such as preventive policy. Indeed, considering Rogers [69]’s note that a distinctive aspect of preventive measures is that “their beneficial effects are (a) delayed in time and (b) difficult to assess” (p. 80), attention to science and environmental news might have led individuals to support policies that can eventually be more beneficial, even though it might take longer. In addition, political news attention was positively related to support for preventive policy as well as punitive policy. While this finding contradicts our prediction, it is possible that political news media, as with other news media, might have led individuals to become aware of environmental issues, increasing involvement with those issues [70]. This involvement might have increased general support for various types of measures to address the problem—but not to the point where the public differentiates the implications of each type of policy. As this potential explanation could be only one among others, future studies could further investigate the factors that may influence the association between specific genres of news media use and policy preferences.

Importantly, the findings of this study indicate that discrete emotions can differentially mediate the impact of news media attention on engagement with risks. When anger or fear results from political news attention, this in turn leads to support for punitive policy to deal with the issue. However, when people feel anxious as a response to either political or scientific/environmental news media use, this leads to increased support for both types of policy to address the problem. These results highlight that not only the genres of news media but also the types of emotion should be taken into account to understand the effects of specific news media on public attitudes and engagement with risks. 

All in all, the present study suggests that news media and journalists should be cautious about how to deal with important science or environmental issues. For example, when political news emphasizes “an overwhelming physical danger” [19] associated with the risk and describes it as being beyond one’s control [20], excessive fear is likely to follow—leading the public only to support policies that can have immediate effects (rather than policies that have long-term beneficial effects) or leading to disengagement with the issue [19,71]. In this sense, it is important to note that the Extended Parallel Process Model [72] underscores the role of both “perceived threat” and “efficacy” in effective risk communication. If media primarily convey a threat of an environmental health risk and the attribution of responsibility, but not information about how individuals can take actions to address the problem, this is likely to result in negative emotions that inhibit the motivation to engage proactively with the problem. 

### Limitations

Despite the theoretical and practical importance of this study, it is important to consider the limitations of our study. First, as this study is based on a single survey and focuses on one issue in the domain of science/the environment, the findings of this study should be interpreted with caution regarding its generalizability for different issue domains or contexts. For example, the distinct role of emotions shown in this study may be shown differentially across issues with varying levels of uncertainty [73]. In addition, the relatively low response rate of the survey warrants special attention as this could have led to a biased sample. Considering that high response rates are an essential part of research for generating reliable and valid results for a survey, future research could use a more systematic survey design that can ensure high levels of response quantity and quality. The present study did not examine the contents of the news coverage. While the main goal of this study was to examine how attention to different news media genres is associated with discrete emotions toward a crisis and thus preferences for different policy initiatives, future studies that investigate the influence of specific contents on discrete emotions about given issues will provide further insights into how the use of specific genres of news media elicits such emotions. 

In a related way, the present study examined attention to science and politics in general, not directly measuring attention to this issue in science and political news. While this was due to the focal interest of this study—examining the impact of attending to science or political news in general, future studies that directly measure attention to this issue will expand our understanding of how news attention shapes public understanding and judgment about social issues. Lastly, as with other survey-based studies, this study could not address the causal influence of news media attention on emotion and policy support. For example, it is possible that people who feel certain emotions are more likely to attend to science or political news. While the present study focused on examining the influence of news media on emotional responses and judgments—based on the theoretical framework provided by theories of emotion and research on an affective mediation model of media effects, future research could employ an experimental or panel design to help clarify the causal relationships suggested in this study. While this study took an initial step toward understanding the relationships among specific news media attention, emotion, and public responses to environmental health risks, future research that takes into account these limitations could provide a clearer picture of how news media can influence public engagement with important social issues through discrete emotional reactions. 

## 6. Conclusions

The purpose of this study was to investigate the relationships among specific news media attention, emotion, and public responses to environmental health risks. The findings demonstrate that news media attention indeed exerts distinct influences on the way people feel about an environmental risk, thus affecting policy preferences. Specifically, our study shows that attention to science news is associated with greater anxiety and anger about the issue, whereas attention to political news is associated with fear and sadness/depression, in addition to anxiety and anger. Furthermore, it was shown that the impact of science news attention on support for preventive policy was mediated by anxiety, whereas the impact of political news attention on punitive policy support was mediated by anger and fear. Overall, our study illustrates that news media should be cautious about how to deal with important public crises since it not only affects the way people feel about the risk but also impacts their judgment about how to address the risk. Despite the unique contributions of this study, it is important to consider its limitations. In particular, considering that this study is based on a single survey on a single issue, future research needs to be conducted with more diversified samples across different types of issues. Research that addresses the limitations of the present study could help clarify our understanding of the relationship between news media use, discrete emotions, and public engagement with risks. 

## Figures and Tables

**Table 1:** Correlation between variables.

Variables	SNA	PNA	Anger	Fear	Anxiety	Sadness	Preventive Policy	Punitive Policy
SNA	__							
PNA	0.61 ***	__						
Anger	0.23 ***	0.24 ***	__					
Fear	0.20 ***	0.24 ***	0.65 ***	__				
Anxiety	0.28 ***	0.26 ***	0.65 ***	0.61 ***	__			
Sadness	0.19 ***	0.22 ***	0.66 ***	0.76 ***	0.51 ***	__		
Preventive policy	0.34 ***	0.36 ***	0.36 ***	0.34 ***	0.44 ***	0.31 ***	__	
Punitive policy	0.25 ***	0.26 ***	0.43 ***	0.42 ***	0.41 ***	0.38 ***	0.78 ***	__

Note. SNA = Science news attention; PNA = Political news attention. The effects of age, gender, education, political orientation, perceived threat, and interest in the fine dust problem on these variables were controlled in the analyses. *** *p* < 0.001.

**Table 2:** Indirect effects of news attention on policy preferences through emotional responses.

Paths	Estimate	*SE*	CI
Science news attention → Anxiety → Preventive Policy	0.08 *	0.02	0.050 to 0.120
Political news attention → Anger → Punitive Policy	0.08 *	0.02	0.031 to 0.110
Political news attention → Fear → Punitive Policy	0.08 *	0.02	0.030 to 0.092
Political news attention → Anxiety → Preventive Policy	0.07 *	0.02	0.024 to 0.087

Note. Estimates are standardized coefficients. * *p <* 0.05.

## Data Availability

Data are available on request due to ethical issues.
